# Double direct hernia, triple indirect hernia, double Pantaloon hernia (Jammu, Kashmir and Ladakh Hernia) with anomalous inferior epigastric artey: Case report

**DOI:** 10.1016/j.ijscr.2019.05.035

**Published:** 2019-06-04

**Authors:** Imtiaz Wani

**Affiliations:** Department of Surgery, DHS, Srinagar, Kashmir, India

**Keywords:** Hernia, Direct, Indirect, Pantaloon, Romberg’s, Saddle, Inferior, Epigastric

## Abstract

**Introduction:**

Double direct and triple indirect type hernia in unilateral inguinal hernia is unique. Anomaly of pathway traversed by inferior epigastric artery is always of surgical importance. Meticulous exploration of the groin is mandatory for diagnosis of rare hernia.

**Case report:**

A 46 year old male presented with the right groin swelling. An inguinal hernia having two direct types separated by anamalous inferior epigastric artery and three indirect types of hernia were present. There was one bubonocele and two funicular types of indirect type of hernia present on the single cord. Anamalous inferior epigastric artery lying superficially at midpoint of inguinal canal was buttresed in the posterior wall to prevent its entrapping in mesh. Individual ligation of the indirect sacs and Lichtenstein repair was done.

This is first kind of unique case report with the new type of hernia reported in the English literature after searching Pubmed, Medline, Google Scholar and Embase database. This new hernia, Double Pantaloon hernia, as a mark of respect and unity, is named after its State of origin, “Jammu, Kashmir and Ladakh” so called as “Jammu Kashmir and Ladakh Hernia”.

**Conclusion:**

Double direct and triple indirect type hernia in a unilateral inguinal hernia is unique. Presence of multiple hernia sacs in the inguinal hernia is a risk for recurrence, if not detected. Inferior epigastric artery in inguinal canal may traverse an aberrant course.

## Highlights

• First kind of case report in literature.
• A unique case with double direct and triple indirect type of inguinal hernia.
• Named as Jammu Kashmir and Ladakh Hernia.
• Inferior epigastric artery may traverse an anomalous course in inguinal canal.
• Presence of multiple hernia sacs ,if undetected, is risk for recurrence.

## 1 Introduction

The exact diagnosis of inguinal hernia is usually made during its repair. A careful exploration of groin is diagnostic in identifying multiple unilateral hernias [1,2]. Indirect inguinal hernia is considered to be of congenital origin. Knowing peculiar type of hernia reduce the risk of inferior epigastric vessels injury and lowers the rate of recurrence [3]. Normally the sac with its contents lies anterior or antero-lateral to cord. Direct inguinal hernias are acquired and the sac protrudes through posterior wall. A rare type of hernia exists called as Pantaloon or saddle-bag hernia or Dual hernia or Romberg’s hernia. This hernia is a combination of indirect and direct sacs on both sides of IEA. There is an obvious direct inguinal hernia and a small indirect type in Pantaloon Hernia. A missed small indirect sac in Saddle bag hernia is a common cause of recurrence [4]. IEA along with vein follows usual course in subperitoneal tissue, and an aberrant course lying outside peritoneum with deviation from normal path traversed is rare. Congenital origin of Inguinal hernia contributes to occurrence of multiple hernia types in the inguinal hernia.

The work has been reported in line with the SCARE 2018 Criteria [5].

## 2 Case presentation

**Fig. 1:**
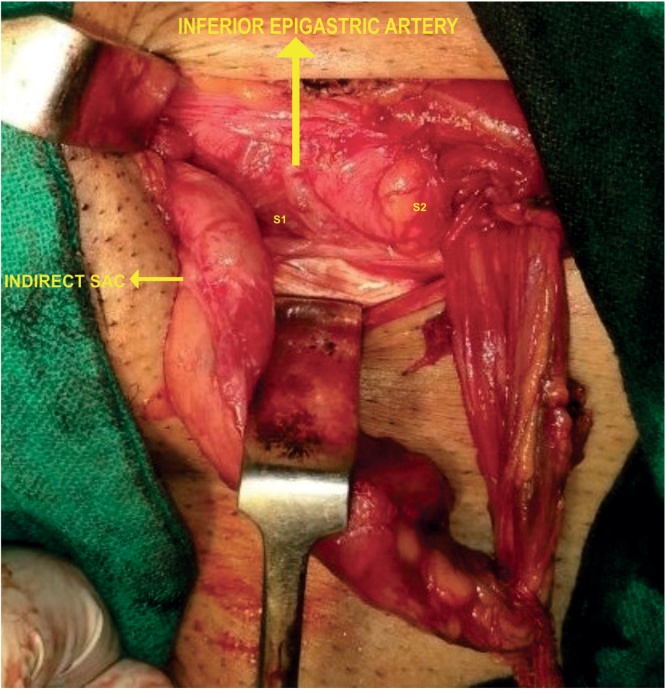
Showing Inferior epigastric artery traversing superficial course in posterior wall of inguinal canal, with two direct sacs, S1 & S2 seen on either side of IEA.

**Fig. 2:**
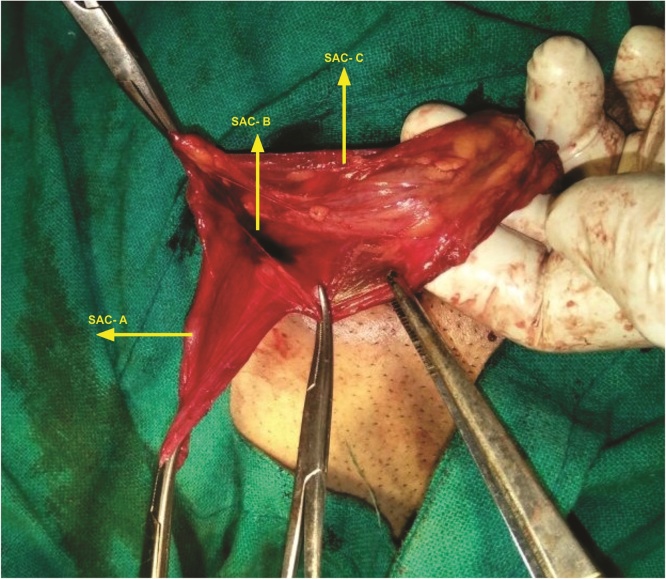
Showing indirect sacs, an unopened sac (A) and the two sacs (B & C) abutting each other with contents.

**Fig. 3:**
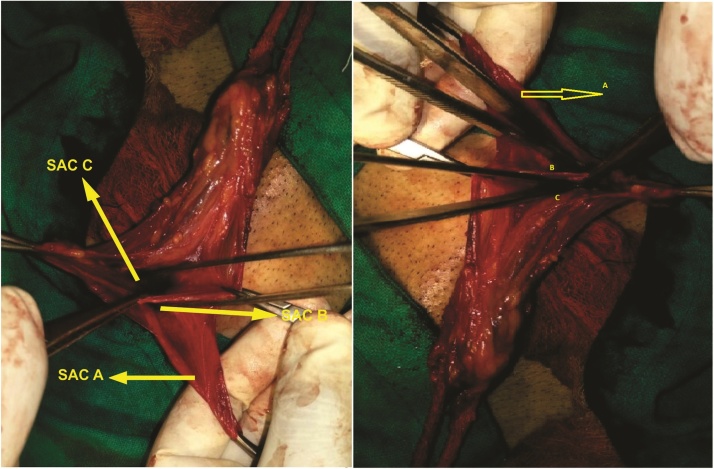
Showing three adjacent indirect sacs adjacent to each other, one unopened sac (A), two indirect sacs (B & C).

**Fig. 4:**
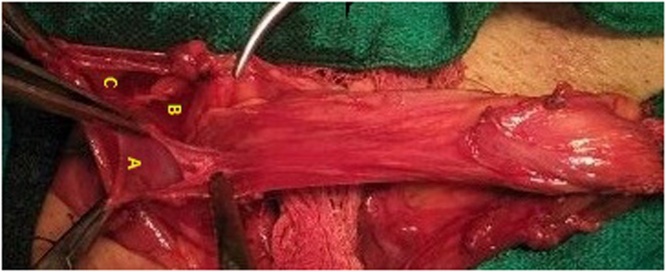
Showing opened all indirect sacs, A, B and C.

A 46 year old male evaluated for right groin swelling had diagnosis of inguinal hernia. There was no history of chronic cough, LUTS or any debilitating disease. Laboratory parameters were normal. Ultrasonography abdomen was normal and of the scrotum confirmed the inguinal hernia. Patient was planned for mesh hernioplasty. On exploration of groin, no inferior epigastric artery was located at medial margin of the deep inguinal ring. The inferior epigastric artery was pursuing anamalous pathway having normal anatomical origin from external iliac artery and piercing rectus sheath. Inferior epigastric artery was traversing superficially on posterior wall, midway between the deep and superficial ring (mid point of inguinal canal) with visible pulsations present [Fig. 1]. A direct component of hernia was seen protruding, one lateral to IEA and another medial to IEA with no cross fluctuation present and each having individual cough expansibility and reducibility [S1 & S2, Fig. 1]. Direct Sac 2 was larger and having wider neck than Sac 1. On exploring the cord, three indirect type of hernia were found on a single cord and few small preperitoneal fatty herniation were seen. There was one bubonocele and two funicular type of indirect hernia present [Fig. 2]. Sac A, Bubonocele, had no content, Sac B funicular type and Sac C, Funicular type had omentum as the content. All three indirect types had individual sac with its peritoneal opening. Sac C, Third sac with omentum as a content was mobilised from cord and was abutting posterior wall of second sac, Sac B [Fig. 3]. Transfixation with ligation of each sac was done individually and released back into the peritoneal cavity [Fig. 4]. IEA was buttresed in normal posterior wall of canal to prevent entrapping of vessel in the mesh. Lichenstein tension free repair with single mesh was done for this double direct hernia. Follow up period of 23 months was normal.

## 3 Discussion

An Indirect inguinal hernia is usually a congenital. A patent processus vaginalis and increased cumulative mechanical exposure are risk factors for indirect inguinal hernia occurrence [6]. Aberrant hernia has been suggested to occur due defective regulatory mechanism of hormones, peptides from the genitofemoral nerve and insufficient release of calcitonin gene-related peptide that have an effect on testicular descent [7].

Landmark point for demarcation of hernia into direct or indirect type is an IEA. Hernia occurring on medial side of IEA is a direct type, whereas those lateral to this is indirect type. IEA is usually located in the area between 4 and 8 cm from midline [8]. IEA originate from external iliac artery just above 5 mm inguinal ligament normally, traverse inguinal canal in subperitoneal, under tranversalis fascia, back to the interfoveolar ligament along medial edge of deep inguinal ring coursing upwards, pierces transversalis fascia and passing infront of linea semicircularis, ascends between rectus abdominis and posterior lamella of its sheath. A great variation with regard to the IEA in relation to its course is observed [9,10]. Clarifying the site of the sac appearance decrease the chance of inferior epigastric vessel injury [3]. There is no documented case where IEA was lying outside tranversalis fascia, superficially on posterior wall of inguinal canal. Placing onlay mesh with this aberrant superficial course of IEA imparts risk of being getting entrapped in mesh and subsequent torrential hemmorhage [11].

Normally, this vessel is located at medial margin of deep ring but location at mid inguinal point is a rarity. This aberrant superficial path traversed by IEA produces two individual sacs of direct hernia on its either side. Both hernia sacs had individual cough expansibility and reducibility with no cross fluctuation.

Lloyld et al. reported occurrence of two cases of ‘third kind of hernia’ with defect lying between the deep ring and inferior epigastric vessels [12]. In contrast to present case, they had two sacs in one case and one direct hernia (third type) in second case. Their findings were demonstrated via TAPP approach, this present case had an open surgery. This third kind of hernia existing with direct hernia reported by Lloyld et al. [12] was corrected to be supravesical hernia coexisting with direct hernia [13]. The present case had an anomalous course pursued by IEA which was lying superficially with two individual direct sacs on either side of that vessel. Clinical significance in this scenario allows sometimes deep ring to be widened on medial side whenever required, otherwise this approach is contraindicated.

Two sacs in unilateral indirect hernia is till date reported in one case only, that too reported in a pediatric case [14]. This case was unique, three indirect type hernia on unilateral side were present, all opening individually with variability in contents of each sac. Congenital anatomical variation during development may lead to this anomaly.

## 4 Conclusion

Double direct and triple indirect type of hernia in a unilateral inguinal hernia is unique. Presence of multiple hernia sacs in an inguinal hernia is the risk for recurrence, if not detected. Inferior epigastric artery in inguinal canal may traverse anomalous course. Careful exploration of the groin is mandatory in diagnosis of unique inguinal hernia.

## Conflicts of interest

None.

## Sources of funding

None.

## Ethical approval

The publication of my article, if the study is exempt from ethnical approval in my institution.

## Consent

Written and signed consent to publish a case report obtained from patient.

## Author contribution

IW made study concept or design, data collection, data analysis or interpretation, writing the paper.

## Registration of research studies

NA.

## Guarantor

Imtiaz Wani.

## Provenance and peer review

Not commissioned, externally peer-reviewed.
